# Correlation of Foraminal Area and Response to Cervical Nerve Root Injections

**DOI:** 10.7759/cureus.286

**Published:** 2015-07-20

**Authors:** Wilson Z Ray, Syed Akbari, Lubdha M Shah, Erica Bisson

**Affiliations:** 1 Neurological Surgery, Washington University School of Medicine in St. Louis; 2 School of Medicine, Washington University School of Medicine in St. Louis; 3 Department of Radiology, University of Utah; 4 Department of Neurosurgery, University of Utah

**Keywords:** cervical spine, selective nerve root block, neuroforamen, cervical radiculopathy, pain, stenosis

## Abstract

*Introduction:* Patients with age-related degenerative changes in the cervical spine leading to cervical spondylosis may be symptomatic or asymptomatic. Older patients with radicular pain tend to have a better response to epidural steroid injections, but it is often difficult to predict which patients will have a positive response to selective nerve root block (SNRB). We analyzed whether the cervical neuroforaminal area measured on MRI predicts immediate therapeutic responses to SNRB in patients who have cervical radiculopathy.

*Methods: *We retrospectively reviewed all patients who had cervical SNRBs treated at a single tertiary referral center. We recorded patient demographics, the neuroforaminal area of the symptomatic and contralateral sides, Visual Analog Scale (VAS) score pre- and post-injection, history of previous cervical surgery, comorbidities, and history of tobacco use.

*Results: *Sixty-four patients with symptoms of cervical radiculopathy treated with neuroforaminal nerve root injections had appropriate imaging and VAS scores recorded. The average foraminal area of the symptomatic side before treatment was significantly smaller than the contralateral asymptomatic neuroforamen (p<0.0001). Those patients with the smallest neuroforamen had a positive response to SNRB. Diabetes and tobacco use did not influence patient response to treatment*.*

*Conclusions: *Measurement of neuroforaminal areas on MRI may represent a useful pre-procedural technique to predict which patients with symptoms of cervical radiculopathy secondary to foraminal stenosis are likely to respond to selective nerve root injections. The predictive ability appears to be limited to those patients with severe stenosis and was less useful in those patients with moderate or mild stenosis.

## Introduction

Age-related degenerative changes in the cervical spine leading to cervical spondylosis are commonly observed in both symptomatic and asymptomatic patients. Spondylotic changes can lead to a broad range of overlapping symptoms, including axial neck pain, cervical radiculopathy, cervical myelopathy, or myeloradiculopathy, although corresponding changes on magnetic resonance imaging (MRI) do not always exist. Currently at our institution, some patients with symptomatic spondylotic changes are initially treated with a diagnostic/therapeutic selective nerve root block (SNRB). While many patients have a very good response to the injection, there remains a large subset of patients that, despite correlating MRI and clinical findings, fail to have a positive response to the injection. The efficacy of lumbar SNRBs remains unconfirmed, with mixed reports in the literature [1-2], and studies investigating the efficacy of cervical nerve root injections and parameters that influence response to injection are scarce.

Previous work has suggested older patients with radicular pain tend to have a better response to both transforaminal and interlaminar cervical epidural steroid injections [3-5], but it is often difficult to predict which patients will have a positive response to SNRB. A surgeon's personal experience and the characteristics of the patient’s spine on MRI (e.g., hard disc, soft disc, osteophytes) also weigh significantly on who is referred for nerve root injection, and little literature exists regarding the role of the neuroforaminal area in clinical response. We hypothesized that patients with foraminal areas below a critical threshold in size would not have a good response to injection because of limited anesthetic/steroid penetration. The purpose of this study was to assess whether the cervical neuroforaminal area measured on MRI can be used to predict immediate therapeutic responses to cervical SNRB in patients with symptoms of cervical radiculopathy.

## Materials and methods

After receiving approval from the University of Utah Institutional Review Board (protocol #53330) with a waiver of informed consent, we undertook a retrospective review of patients who had undergone a cervical SNRB over a two-year period (January, 2009–December, 2010) treated at our tertiary referral center. Inclusion/exclusion criteria included patients referred to our department for surgical evaluation of symptoms of cervical radiculopathy (motor, sensory, or pain), an MRI ≤ 6 months old available for direct review, no findings of cervical myelopathy, and no history of previous cervical surgery. All patients had a clinical history of cervical radiculopathy with MRI findings corresponding to physical examination findings. No patients with cervical myelopathy or purely axial neck pain were included in the analysis. Only patients receiving injections for a single level were included. We collected patient demographics, the Visual Analog Scale (VAS) score pre- and post-injection, history of previous cervical surgery, comorbidities, and history of tobacco use.

All SNRBs were done with fluoroscopic guidance using a biplane fluoroscopic unit. The patient was placed in the supine position. The vertebral and facet joint margins were aligned under fluoroscopy. The target was the superior articular process of the same number vertebra as the nerve being injected. Approximately 1 mL of 2% lidocaine was used for local analgesia. The needle was then deflected anteriorly into the neural foramen. Approximately 2 mL of myelography-safe iodinated contrast agent was used for the needle tip confirmation in the nerve root sleeve. The contrast pattern was identified as intraneural, perineural, and, most importantly, not intra-arterial. The injectate used in these procedures was 1 mL of 10 mg/mL dexamethasone and 1 mL of 1% lidocaine. We used nonparticulate steroids (dexamethasone) for our neural foraminal injections because of the reported risk of embolic infarctions due to inadvertent intra-arterial injection of particulate steroids [6-7]. A post-procedure VAS score was obtained 30 minutes after the injection by the treating neurointerventionalist.

**Figure 1:**
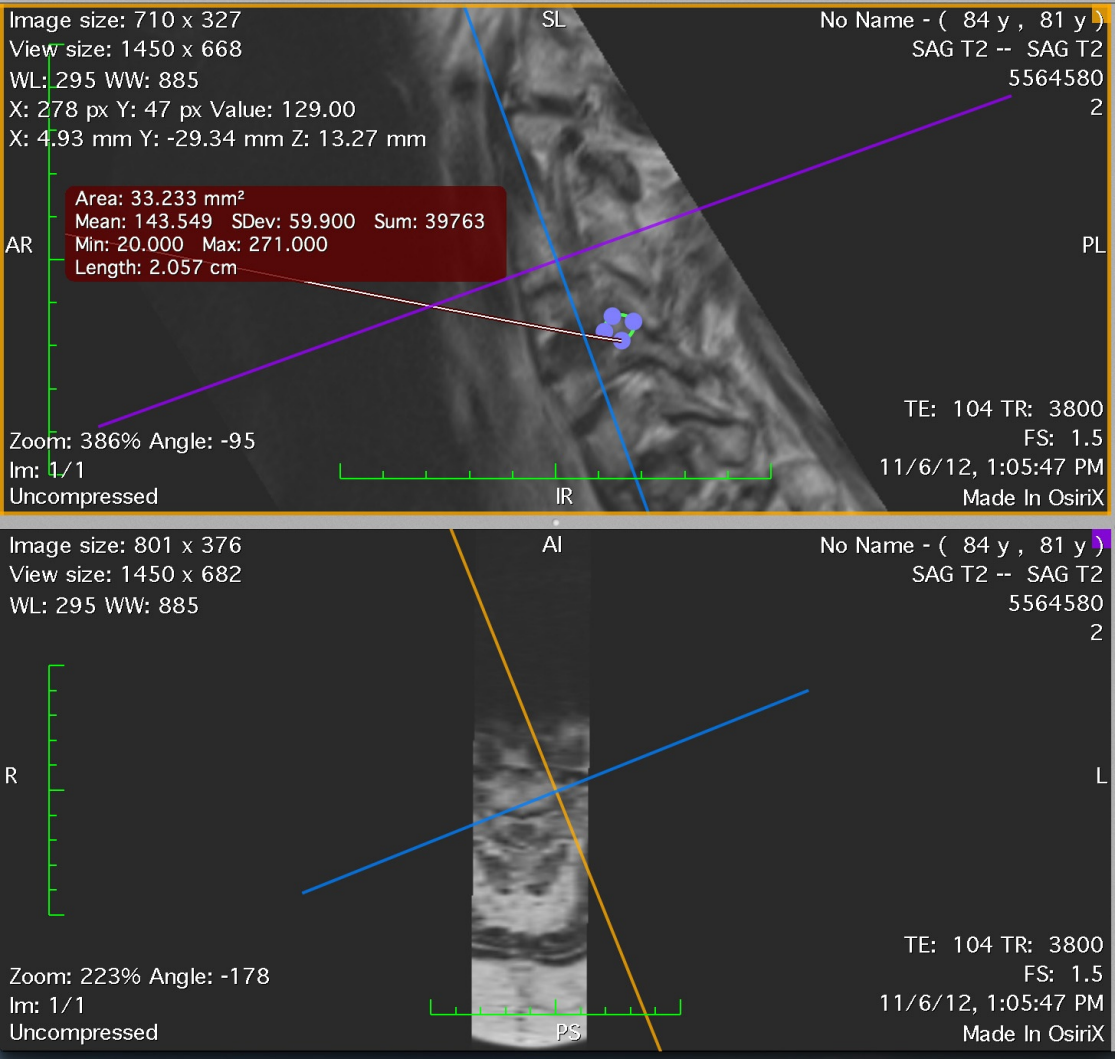
The region of interest tool was used to calculate the maximum dimension of the neural foramen in the oblique sagittal plane.

We measured the neuroforaminal area of the symptomatic side, using the asymptomatic contralateral side as the internal control. Measurements of neuroforaminal areas were made by both a spine neurosurgeon and a neuroradiologist blinded to patient VAS scores. Measurements were performed on the MRI Digital Imaging and Communications in Medicine (DICOM) data set, imported into the Osirix software (version 3.8.1 32-bit). The sagittal T2-weighted sequences were selected and post-processed with the three-dimensional multi-projection reformat program. Using the crosshairs, maximal neuroforaminal dimensions were evaluated at the level of interest in the oblique sagittal plane by each observer independently. The region of interest tool was used to calculate the maximum dimension of the neural foramen in the oblique sagittal plane (Figure 1).

### Statistical analysis

Statistical analyses were performed using a Student *t*-test for paired ipsilateral and contralateral foraminal area. Analyses involving continuous variables across four groups were performed using ANCOVA, with diabetes and smoking status as covariates. The analyses were performed using SPSS. All tests were two-sided, and a P-value <0.05 was used to indicate statistical significance.

## Results

Sixty-four patients were identified for inclusion in this study. The average age of the subjects in the cohort was 48.1 ± 8.9 years (range: 24–70 years). The distribution by sex was approximately equal (Table 1). Twenty percent of patients were current or former smokers. A small minority of patients were diabetic (6.3%), with the most common comorbidity being hypertension (15%). The most commonly treated level was C5/6 (29 patients, 45.3%); 24 (37.5%) patients were treated at C6/7, and the number of patients treated at other levels was much lower.

**Table 1:** Characteristics of patients treated with cervical selective nerve root block

	N = 64 (%)
Sex	
Female	31 (48.4)
Male	33 (51.6)
Number of patients treated at each spinal level (%)	
C3-4	2 (3.1)
C4-5	4 (6.3)
C5-6	29 (45.3)
C6-7	24 (37.5)
C7-T1	5 (7.8)
Smoking status	
Current	10 (15.6)
Former	3 (4.7)
Never	51 (79.7)
Diabetes	
Yes	4 (6.3)
No	60 (93.8)
Comorbidities	
Heart disease	2 (3.1)
Hypertension	10 (15.6)
Chronic obstructive pulmonary disease	1 (1.6)

The measurements of the foraminal area between the two reviewers were averaged for the purpose of our analysis. The range of deviation between the two reviewers for each subject was -0.227 to 0.182 cm^2^, with an average deviation of 0.001 cm^2^. The reviewers were not significantly different in their measurements of foraminal area (p=0.829, paired t-test).

The average foraminal area of the side ipsilateral to injection (0.154 ± 0.118 cm^2^) was significantly smaller than the contralateral asymptomatic side, respectively (0.224 ± 0.113 cm^2^) (*P=*0.0001). Mean pre-procedural VAS score for all patients was 5.48 ± 2.23 (range: 1–10) and mean post-procedural VAS score (30 minutes after the procedure) was 1.91 ± 2.65 (range: 0–10). The average improvement in VAS for all patients after injection was 3.58 ± 2.68 points. An excellent response to SNRB was defined as complete resolution of arm pain, a good response was defined as at least a 50% reduction in arm pain, and a fair or poor response was defined as a less than 50% reduction in arm pain or no response to injection [8]. Forty-three of the 64 patients had a good/excellent response to injection (67%). Ten patients (16%) had a fair response to injection, nine patients (14%) had no response to injection, and two patients (3%) reported higher VAS scores after injection.

Table 2 provides a summary of clinical outcomes based on the degree of foraminal stenosis. The overall range of foraminal areas was 0.0145 to 0.5005 cm^2^.

**Table 2:** Mean change in VAS score by degree of stenosis as measured by foraminal area

Area range (cm^2^)	n	Mean VAS benefit	p-value
0.0145 – 0.1360	35	3.80 ± 2.52	0.140
0.1370 – 0.2575	17	3.18 ± 2.60	
0.2576 – 0.3790	8	2.25 ± 3.45	
0.3800 – 0.5005	4	6 ± 0.81	

The foraminal areas were arbitrarily divided into four equal ranges, with the majority of patients falling into the group with the smallest neuroforaminal areas. In the groups with the most stenosis, we observed a trend in VAS scores with those patients with the highest degree of stenosis (i.e., the smallest foraminal area), having the greatest response to injection. Despite this initial trend, Group 4, those patient's with minimal foraminal stenosis 0.379–0.5005 cm^2^ (n=4), also reported a significant improvement in VAS score after injection. The level of benefit between the four groups was not statistically significant (p=0.140). None of the patients had been treated with oral methylprednisolone. Diabetes and smoking status were not statistically significant predictors of patient response to injection.

## Discussion

Our results suggest that patients with severe neuroforaminal narrowing benefit from cervical SNRB. While our study involved only a small group of patients, our results did not support our initial hypothesis. We had anticipated finding a subset of patients with very small foraminal areas that would not respond SNRB. Our hypothesis was based on the idea that there would be a critical stenosis or foraminal area that was too small to allow adequate steroid/anesthetic penetration. In contrast, our results suggest that patients with very small neuroforaminal areas are likely to have an immediate response to injection while patients with mild to moderate stenosis are less predictable. Interestingly, we also observed four patients with minimal foraminal stenosis that reported a dramatic improvement in VAS scores (6 ± 0.81) after SNRB. An excellent response was consistently reported in all four patients. One hypothesis to explain the outcome for these patients is that neural foraminal narrowing may be occurring in the dynamic flexion/extension position, which is not assessed on the standard static supine MRI [9]. An alternative explanation would be that these four patients had a more chemical radiculitis because of a tear in the annulus and spread of inflammatory cytokines into the epidural space. This may explain their symptoms of radiculopathy without an appreciable disc herniation [10-11] and may explain why these patients experienced a favorable response despite minimal foraminal narrowing.

Cervical SNRBs are often used as both a diagnostic and a therapeutic adjuvant in the management of degenerative cervical spondylosis [12-13]. While there is still considerable debate about whether local anesthetic alone or a combination of local anesthetic and corticosteroids is more effective [14-19], SNRBs have been demonstrated to be a useful treatment option in patients with both degenerative cervical and lumbar spondylosis. Multiple authors have suggested that patients older than 50 years of age with radicular symptoms respond better to cervical epidural steroid injections, yet often anecdotal experience weighs heavily on patient selection [3-4, 20]. Although positive imaging findings suggestive of neuroforaminal compromise provide some guidance in patient selection [21], MRI findings have not been reliably shown to be predictive of a positive response to SNRB, and there is little or no literature on how cervical foraminal area correlates with patient response to SNRB.

The use of interlaminar and transforaminal epidural steroid injections in the lumbar spine have been well described in the literature [1-2]. Less literature is available regarding the efficacy of injections in the cervical spine [12-13, 15], with only a few prospective randomized trials reported to date [14, 22]. Bush, et al. [22] prospectively studied 68 patients with symptoms of cervical radiculopathy treated with serial epidural or periradicular corticosteroid injections. At an average of 39 months follow-up, 76% (48 patients) had resolution of radicular symptoms without any surgical intervention. More recently, Anderberg, et al. [14] prospectively reviewed a series of 40 patients with cervical radiculopathy that underwent SNRB with either a combination of corticosteroids and local anesthetic or local anesthetic alone. A three-week follow-up time point revealed no significant difference between the treatment groups. The authors suggest steroids may not provide any additional benefit in patients undergoing SNRB for symptoms of cervical radiculopathy.

MRI findings of cervical spondylosis are exceedingly common after the age of 50 years of age [23-24], and while it is common to have MRI findings of spondylosis without corresponding clinical symptoms, it is also common to encounter the opposite with minimal radiographic findings and strong subjective clinical complaints. Interpreting the MRI findings and correlating these to patient complaints can be challenging; however, Strobel, et al. [21] demonstrated that the most important MRI finding predictive of response to transforaminal epidural steroid injection is neuroforaminal involvement. They retrospectively reviewed the results of 93 patients treated with transforaminal epidural steroid injections to determine whether MRI findings could predict response to treatment. The authors assessed pre- and post-procedure VAS scores in patients treated with a combination of local anesthetic and corticosteroids and found that foraminal disc herniation (*P*=0.034), foraminal nerve root compromise (*P=*0.013), and the absence of spinal canal stenosis (*P=*0.013) were statistically significant predictors of a positive response to transforaminal epidural steroid injection and portended a better response to treatment. The results of our study corroborate these findings. There are some inherent limitations with MRI. Image quality may be compromised by section thickness, decreased signal-to-noise ratio due to coil selection and technical parameters, partial volume averaging, and cerebrospinal fluid pulsation artifact; however, in a comparison of MR multi-echo data image combination sequence and CT myelography, Dorenbeck, et al. [25] found no statistical differences between these imaging modalities in the assessment of neuroforaminal or spinal canal narrowing.

## Conclusions

Nerve root injections are an important treatment option in the management of patients with cervical radiculopathy. Although our study population represents only a small subset of patients, our results suggest that those patients with very small neuroforamina secondary to stenosis respond well to injection, while those patients with moderate to mild stenosis are less predictable in their response to injection. While larger prospective studies are needed to verify these results, measurement of neuroforaminal areas on MRI may represent a useful pre-procedural technique to predict which patients with symptoms of cervical radiculopathy secondary to foraminal stenosis are likely to respond to selective nerve root injections.
